# Retraction Note: LncGPR107 drives the self-renewal of liver tumor initiating cells and liver tumorigenesis through GPR107-dependent manner

**DOI:** 10.1186/s13046-022-02414-1

**Published:** 2022-07-25

**Authors:** Guanqun Huang, Hui Jiang, Ye Lin, Wuzheng Xia, Yuanwei Luo, Yanpeng Wu, Weilong Cai, Xinke Zhou, Xianhan Jiang

**Affiliations:** 1 Department of general surgery, The Fifth Affiliated Hospital of Guangzhou Medical University, Guangzhou, China; 2 Department of Abdominal Oncology, The Fifth Affiliated Hospital of Guangzhou Medical University, Guangzhou, China; 3 Department of General Surgery, Guangdong General Hospital, Guangdong Academy of Medical Sciences, Guangzhou, China

**Retraction Note: J Exp Clin Cancer Res 37, 121 (2018)**

**https://doi.org/10.1186/s13046-018-0794-3**

The Editor-in-Chief has retracted this article at the corresponding author's request. After publication, concerns were raised regarding image overlap between Fig. 4g and an article from a different group [1].

The authors provided partial raw data, which was insufficient to address the image overlap concerns. Additionally, the raw data provided for Fig. 6d revealed that the images representing the lncASO and SRCAP KO groups may have originated from a single sample. Similarly, the images representing the two GRP107 KO groups also may have originated from a single sample.

A further investigation by the publisher has revealed that there is no statement of Ethics approval for the use animals in the article. Additionally, in Fig. 6b, a number of data points refer to tumours that weigh 2.8-3 g, which exceed the recommended tumour weight limit of 10% of initial bodyweight. The authors have been unable to retrieve the original animal ethics approval documents. Xinke Zhou has stated on behalf of all authors that none of the authors agree to this retraction.
